# Absence of causal association between Vitamin D and bone mineral density across the lifespan: a Mendelian randomization study

**DOI:** 10.1038/s41598-022-14548-5

**Published:** 2022-06-21

**Authors:** Yanchao Tang, Feng Wei, Miao Yu, Hua Zhou, Yongqiang Wang, Zhiyong Cui, Xiaoguang Liu

**Affiliations:** 1 Department of Orthopaedics, Peking University Third Hospital, 49 North Garden Street, HaiDian District, Beijing, 100191 China; 2 Beijing Key Laboratory of Spinal Disease Research and Engineering, Beijing, China; 3 Research Center of Bone and Joint Precision Medicine, Ministry of Education, Beijing, China; 4 Health Science Center, Peking University, Beijing, China

**Keywords:** Genetic association study, Osteoporosis, Ageing, Bone, Bone quality and biomechanics, Calcium and vitamin D, Genetics research, Risk factors

## Abstract

Vitamin D deficiency is a candidate risk factor for osteoporosis, characterized by decreased bone mineral density (BMD). We performed this two-sample Mendelian randomization (MR) analysis to investigate the causal effect of vitamin D on BMD. We extracted 143 single-nucleotide polymorphisms from a recent GWAS on 417,580 participants of European ancestry as instrumental variables, and used summary statistics for BMD at forearm (n = 10,805), femoral neck (n = 49,988), lumbar spine (n = 44,731) and total-body of different age-stages (< 15, 15–30, 30–45, 45–60, > 60) (n = 67,358). We explored the direct effect of vitamin D on BMD with an adjusted body mass index (BMI) in a multivariable MR analysis. We found no support for causality of 25-hydroxyvitamin D on BMD at forearm, femoral neck, lumbar spine, and total-body BMD across the lifespan. There was no obvious difference between the total and direct effect of vitamin D on BMD after adjusting for BMI. Our MR analysis provided evidence that genetically determined vitamin D was not causally associated with BMD in the general population. Large-scale randomized controlled trials are warranted to investigate the role of vitamin D supplementation in preventing osteoporosis in the high-risk population.

## Introduction

In recent years, osteoporosis and osteoporotic fractures have become a major threat to the health of the elderly with a growing aging population^1,2^. Early identification of clinical risk factors is essential for the prevention and treatment of the disease^3,4^.

Osteoporosis is a chronic disease characterized by bone loss and microarchitecture impairment. Osteoporosis is diagnosed and monitored mainly by the measurement of bone mineral density (BMD) at the central (e.g., the lumbar spine and the femoral neck) and peripheral (e.g., the distal forearm) sites by dual-energy X-ray absorptiometry (DXA)^5–7^. Vitamin D, a fat-soluble vitamin that acts as a steroid hormone, has been identified as a physiological factor associated with BMD^8^. It plays a crucial role in skeletal health by regulating calcium and phosphorus metabolism^9,10^. 25-hydroxyvitamin D (25OHD) is the primary circulating biomarker to assess vitamin D status. Influential advocates argued that a large portion of the Western population was vitamin D deficient and vitamin D supplementation continued to be widely used among older adults^11–15^. However, as bone status varies with age and anatomical location^15,16^, whether the serum 25OHD concentration affects BMD at different skeletal sites or among age groups remains unclear^17–19^.

Two-sample Mendelian randomization (MR) analysis with single nucleotide polymorphisms (SNPs) as instrumental variables (IVs) is used to assess the causal effect of an exposure on an outcome. The two-sample MR analysis can overcome the limitations of observational studies, but its validity relies on the following three assumptions: (1) the genetic variant is associated with exposures; (2) the genetic variant is not associated with confounders; and (3) the genetic variant influences the outcome only through the exposures^20,21^. An increasing number of IVs used in MR are available due to the genome-wide association studies (GWAS) being conducted and high-throughput genomic technologies being developed. Several previous MR studies used SNPs in or near genes (DHCR7, GC, CYP2R1, and CYP24A1) encoding enzymes and carrier proteins involved in vitamin D synthesis or metabolism as instrumental variables. They did not find a causal association between the serum 25OHD concentration and BMD measured at the forearm (FA), lumbar spine (LS), hip, or total body (TB)^22–24^.

In this study, we implement an MR approach to explore the causal effect of vitamin D on BMD values at different skeletal sites and across the lifespan. We employ the most recent GWAS of 25OHD concentration to optimize the power of our two-sample MR analysis. This approach provides an effective overall estimate of the effect of vitamin D on bone health while accounting for bias due to body mass index (BMI).

## Results

### Selection of instrumental variables

We extracted 143 independent SNPs from a recent GWAS on 417,580 participants of European ancestry as IVs to genetically predict one standard deviation (SD) increase in the rank-based inverse-normal transformed (RINT) 25OHD concentration^25^. The distribution of 25OHD concentration was right-skewed and showed the expected seasonal fluctuation, with a median, mean and interquartile ranges of 47.9, 49.6, 33.5–63.2 nmol/L^25^. The detailed characteristics of SNPs are shown in Supplementary Table S1. For these IVs, all F statistics were above 10 (ranging from 30.04 to 8036.12, average 219.41), indicating that they satisfy the first MR assumption and that weak instrument bias would not substantially influence the estimations of causal effects. These genetic variants explained 5.4% of phenotypic variation of the serum 25OHD concentration.

### The combined effect of 25OHD on BMD at different skeletal sites

Summary statistics of beta coefficients and standard errors were drawn from the Genetic Factors for Osteoporosis (GEFOS) Consortium for assessing the impact of the 25OHD-associated SNPs on the forearm (FA), femoral neck (FN), and lumbar spine (LS), BMD (expressed as standard deviations from the mean). After harmonizing the exposure and outcome datasets, 108, 105, and 105 SNPs were involved in FA-BMD, FN-BMD, and LS-BMD MR analyses. The detailed characteristics of SNPs used for assessing the effect of 25OHD on BMD at different skeletal sites are shown in Supplementary Tables S2-4.

**Figure 1:**
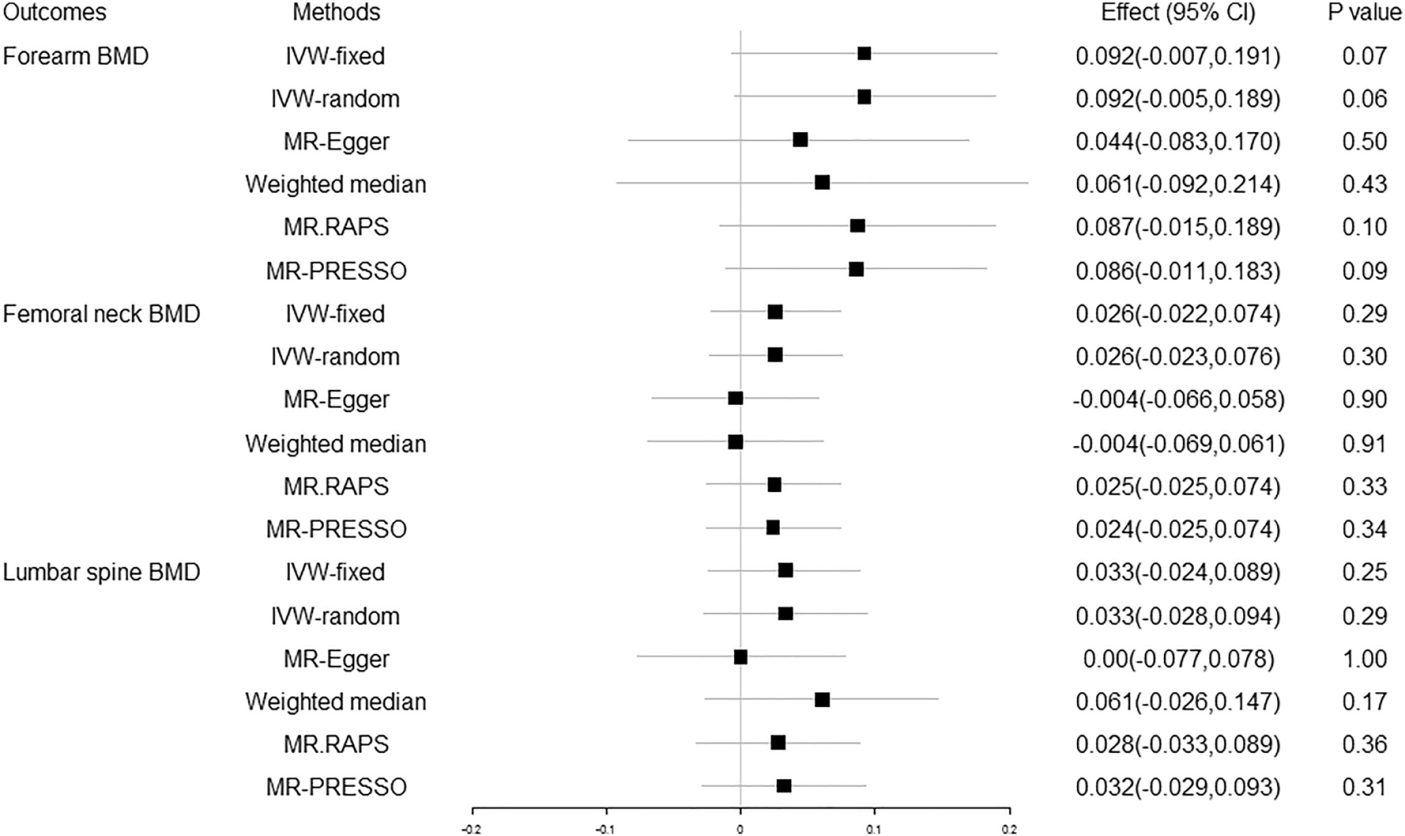
Causal effect of 25OHD on BMD at different skeletal sites. 25OHD 25-hydroxyvitamin D, BMD bone mineral density, the combined causal effect of 25OHD on BMD, CI confidence interval, IVW-fixed inverse variance weighted with fixed effects, IVW-random inverse variance weighted with random effects, MR.RAPS MR Robust Adjusted Profile Score, MR-PRESSO MR Pleiotropy RESidual Sum and Outlier.

The MR estimates from different methods of the causal effect of 25OHD on BMD at FA, FN and LS are presented in Fig. 1 and Supplementary Table S5. No causal effect of increased/decreased serum 25OHD concentration on FA-BMD, FN-BMD, or LS-BMD was found at a Bonferroni corrected p threshold of 0.01.

The heterogeneity was analyzed using the IVW, MR-Egger, and MR-PRESSO methods and the pleiotropy was investigated using the MR-Egger intercept test, as shown in Supplementary Table S6. No evidence of heterogeneity was observed Our analysis suggested no significant evidence of horizontal pleiotropy (as indicated by MR-Egger regression intercept close to zero, with a *P* value larger than 0.05). We performed a “leave-one-out” analysis based on the IVW method and found that there was no potentially influential SNP driving the causal link. The inference was based on a single SNP using the Wald ratio method, the scatter plots and funnel plots for the causal relation between 25OHD and BMD in FA, FN, and LS were stable. No horizontal pleiotropic outlier was identified for FA-BMD, FN-BMD, or LS-BMD in the MR-PRESSO test.

### The combined effect of 25OHD on BMD of different age stages

Summary statistics for the impact of the 25OHD-associated SNPs on total body BMD (TB-BMD) in each of five age strata spanning 15 years, including TB-BMD (> 60), TB-BMD (45–60), TB-BMD (30–45), TB-BMD (15–30) and TB-BMD (0–15) were also drawn from the GEFOS Consortium. After harmonizing the exposure and outcome datasets, 122 SNPs were involved in TB-BMD MR analysis, and 121, 121, 120, 116, 121 SNPs were involved in TB-BMD (> 60), TB-BMD (45–60), TB-BMD (30–45), TB-BMD (15–30) and TB-BMD (0–15) MR analyses. The detailed characteristics of SNPs used for assessing the effect of 25OHD on BMD in different age groups are shown in Supplementary Tables S7–12.

**Figure 2:**
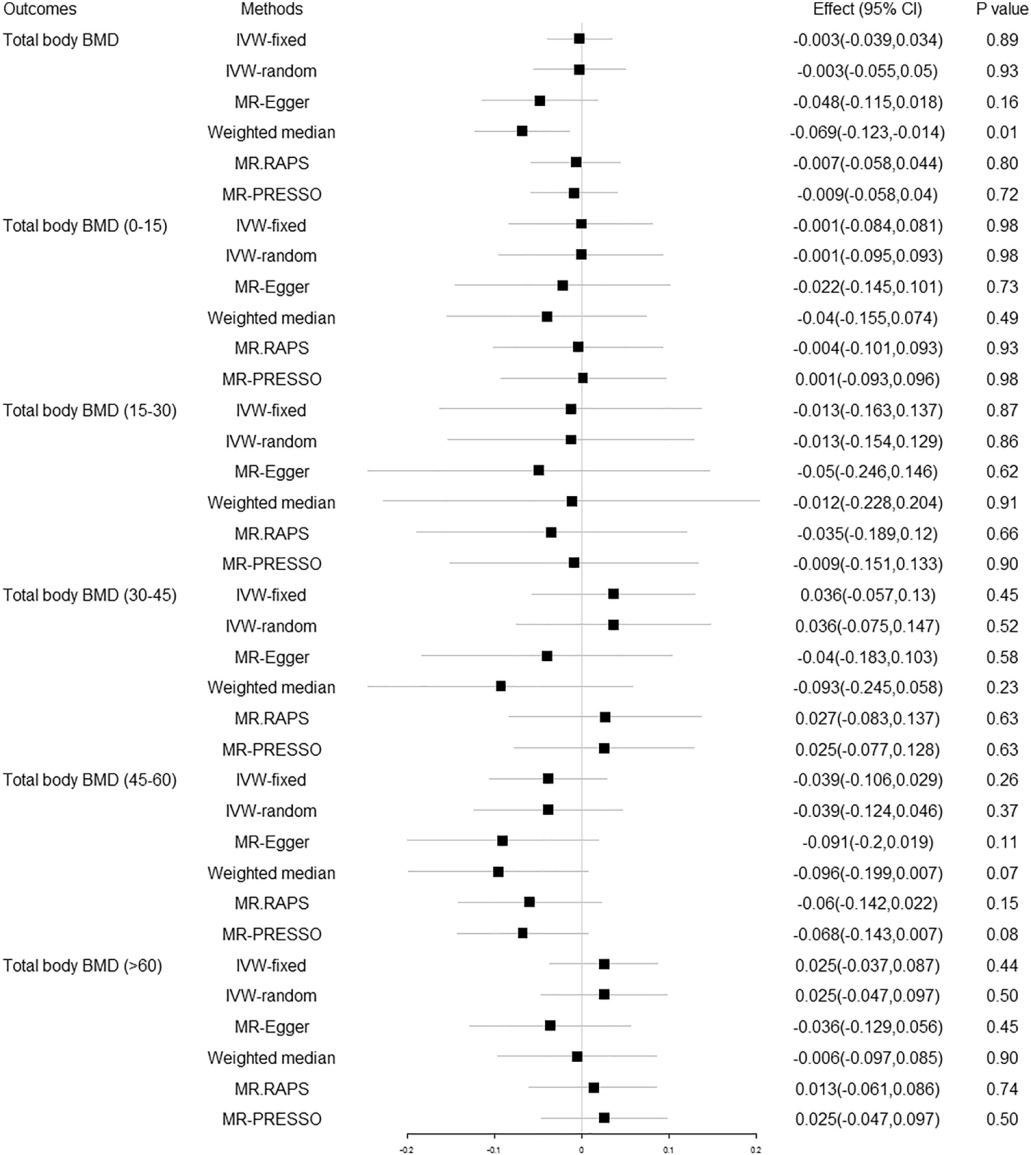
Causal effect of 25OHD on TB-BMD in different age groups. 25OHD 25-hydroxyvitamin D, BMD bone mineral density, Effect the combined causal effect of 25OHD on BMD, CI confidence interval, IVW-fixed inverse variance weighted with fixed effects, IVW-random inverse variance weighted with random effects, MR.RAPS MR Robust Adjusted Profile Score, MR-PRESSO MR Pleiotropy RESidual Sum and Outlier.

The MR estimates from different methods of the causal effect of 25OHD on TB-BMD and TB-BMD in different age groups are presented in Fig. 2 and Supplementary Table S13. No causal effect of increased/decreased serum 25OHD concentration on TB-BMD was found at a Bonferroni corrected p threshold of 0.01.

Heterogeneity analysis using the IVW, MR-Egger, and MR-PRESSO methods highlighted the existence of heterogeneity in TB-BMD, TB-BMD (> 60), TB-BMD (45–60), TB-BMD (30–45), and TB-BMD (0–15) (P < 0.05, Supplementary Table S14 Pleiotropy analysis using the MR-Egger intercept test observed directional pleiotropy in TB-BMD and TB-BMD (> 60) (*P* < 0.05, Supplementary Table S14). We performed a “leave-one-out” analysis based on the IVW method and found that there was no potentially influential SNP driving the causal link. The inference based on a single SNP using the Wald ratio method, the scatter plots, and funnel plots for the causal relation between 25OHD and BMD of different age stages were stable. In MR-PRESSO analysis, we found six outliers for TB-BMD, but the results were similar after removing these SNPs (Distortion test *P* = 0.441).

### Power of analysis

Table 1 shows the sample sizes in the current analysis for the nine BMD traits. We present the power estimations and minimum detectable causal effects for 80% power based on the sample sizes, the proportions of 25OHD variation explained by genetic variants, and the causal effects observed. The statistical powers of age-stratified analyses were lower due to the small sample sizes of each of the age strata. The minimum detectable causal effects were more than 0.05; that is, these analyses were underpowered to exclude causal effects less than 0.05.

**Table 1:** Sample size of BMD groups and statistical power of MR analysis.

Outcome	Sample size	r^2^	Effect	Power	Minimum detectable effect
Forearm BMD	10,805	4.97%	0.0921	56.9%	0.1209
Femoral neck BMD	49,988	4.94%	0.0261	25.4%	0.0564
Lumbar spine BMD	44,731	4.94%	0.0329	34%	0.0596
Total body BMD	56,284	5.18%	− 0.0025	3.4%	0.0519
Total body BMD (0–15)	11,807	5.18%	− 0.0013	2.7%	0.1133
Total body BMD (15–30)	4,180	5.02%	− 0.0128	3.8%	0.1933
Total body BMD (30–45)	10,062	5.17%	0.0362	12.8%	0.1228
Total body BMD (45–60)	18,805	5.18%	− 0.0388	22.7%	0.0898
Total body BMD (> 60)	22,504	5.18%	0.0247	13.2%	0.0821

BMD bone mineral density, MR Mendelian randomization, r2 the proportion of phenotypic variation explained by genetic variants.

The minimum detectable effect per standard deviation increase/decrease in 25-hydroxyvitamin D concentration: assume 80% power and 0.05 significance level.

### Repeated analysis using SNPs in or near vitamin D related genes as IVs

**Figure 3:**
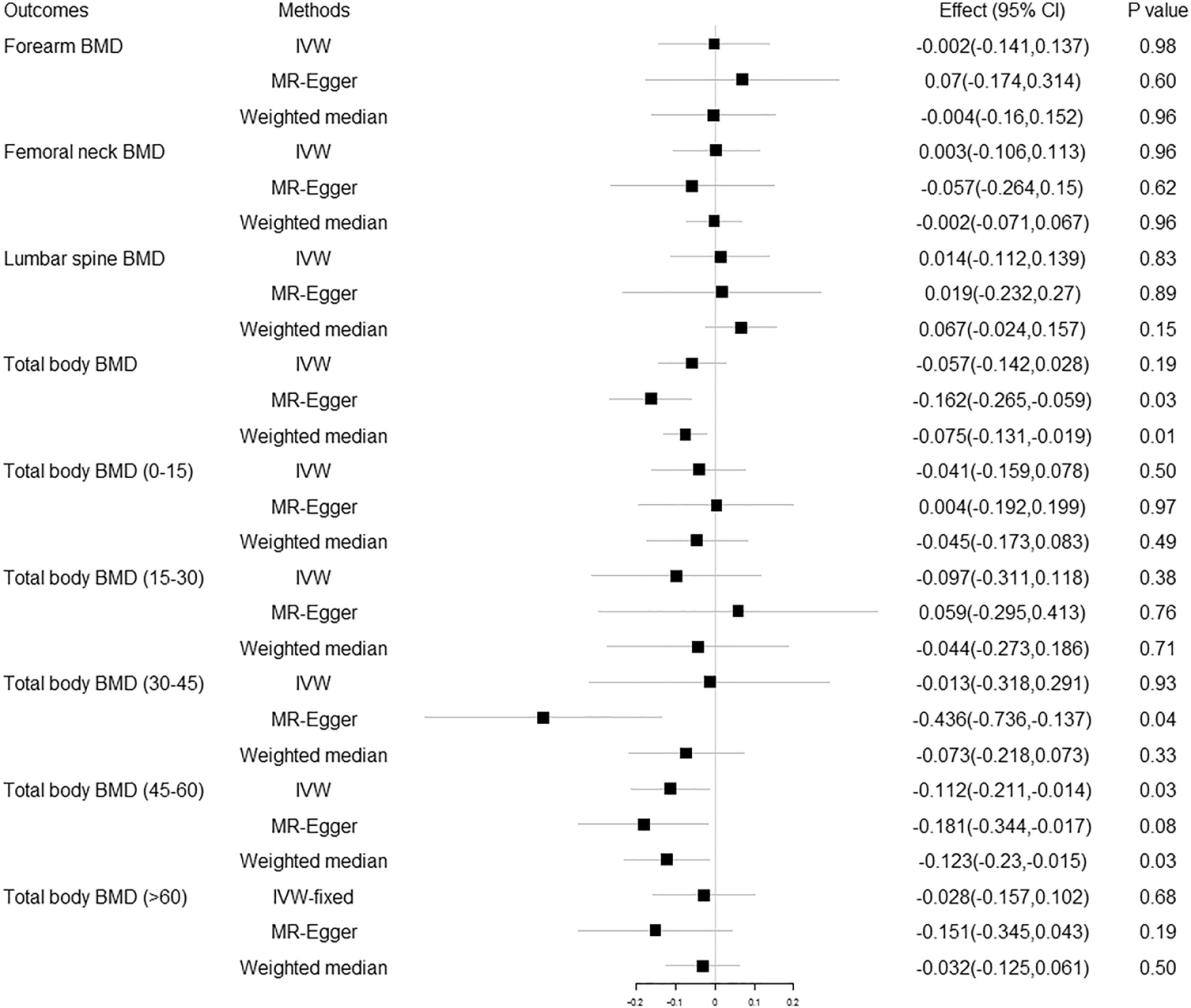
Replication analysis of the causal effect of 25OHD on BMD using SNPs in or near vitamin D related genes as instrumental variables. 25OHD 25-hydroxyvitamin D, BMD bone mineral density, Effect the combined causal effect of 25OHD on BMD, CI confidence interval, IVW inverse variance weighted.

Seven SNPs (GC-rs705117, GC-rs1352846, CYP2R1-rs117576073, CYP2R1-rs12794714, CYP24A1-rs17216707, CYP24A1-rs2585442, CYP24A1-rs2762943) were selected as IVs on the basis of their location in or near previously reported vitamin D genes. The MR estimates from different methods of the causal effect of 25OHD on TB-BMD and TB-BMD in different age groups are presented in Fig. 3 and Supplementary Table S15. No causal effect of 25OHD on BMD was found at a Bonferroni corrected p threshold of 0.01.

### Multivariable MR analysis with BMI

**Figure 4:**
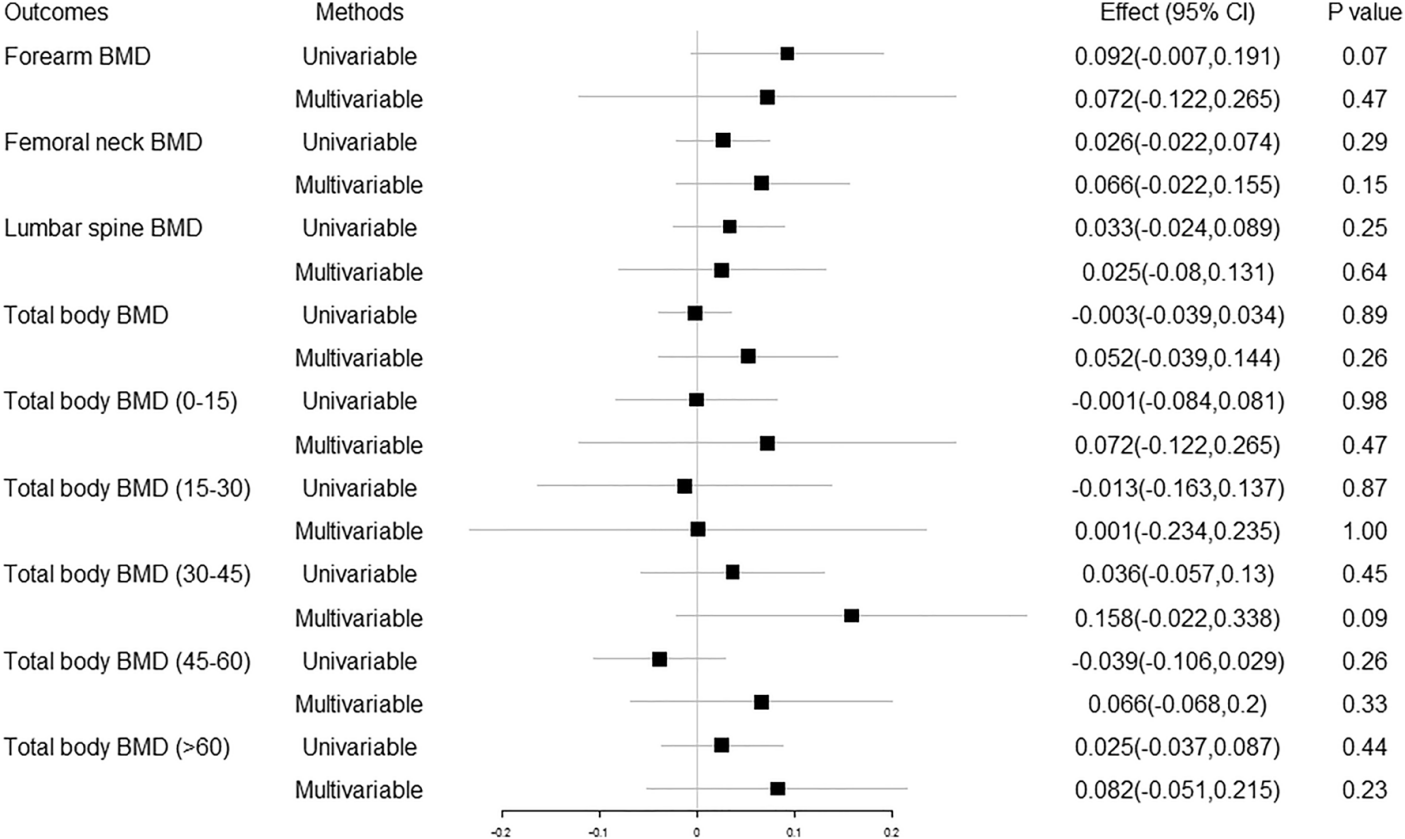
Univariable and multivariable Mendelian randomization analysis of the causal effect of 25OHD on BMD. 25OHD 25-hydroxyvitamin D, BMD bone mineral density, Effect the combined causal effect of 25OHD on BMD, CI confidence interval.

We extracted 122 independent SNPs as IVs to genetically predict one standard deviation (SD) increase in 25OHD concentration and BMD. The detailed characteristics are shown in Supplementary Table S16. The multivariable MR estimates of the causal effect of 25OHD and BMI on BMD at different skeletal sites and across the lifespan are presented in Fig. 4 and Supplementary Table S17. No causal effect of increased serum 25OHD concentration on BMD was found. No significant difference between the total and direct effect of vitamin D on BMD after adjusting for BMI was observed.

### The causal effect of 25OHD on ultrasound-measured heel BMD

We extracted 108 SNPs that were found in the SUNLIGHT (N = 79,366) consortium samples from the 143 genome-wide significant SNPs as IVs (Supplementary Table S1). After harmonizing the exposure and outcome datasets, 107 SNPs were involved in the two-sample MR analysis. The MR estimates of the causal effect of 25OHD on ultrasound-measured heel BMD are presented in Supplementary Table S18. The causal effect was observed only in MR.RAPS method (Effect (95%) CI − 40.1677 (− 69.3513, − 10.9841), *P* = 0.007). Heterogeneity and pleiotropy tests (Supplementary Table S19) observed the existence of heterogeneity (IVW, Q statistic 1012.3, Degree of freedom 106, *P* = 8.87E-148; MR-Egger, Q statistic 1009.5, Degree of freedom 105, *P* = 1.02E−147; MR-PRESSO, Global test 1003.1, *P* < 0.001) and no pleiotropy (*P* = 0.589).

## Discussion

Motivated by the prevalence of vitamin D supplementation among adults, we conducted this two-sample MR analysis to explore the causal relationship between vitamin D and BMD across the lifespan. With the larger sample size, the instruments selected explained approximately 5% of the variance in the serum 25OHD concentration compared with the 1% accounted for in the previous iteration^22,24^. We found no support for causality, which was replicated using SNPs in the previously reported vitamin D genes. In the multivariable MR analysis, no obvious difference between the total and direct effect of vitamin D on BMD after adjusting for BMI was observed, which is solid evidence of the above conclusion.

Serum 25OHD is a fat-soluble hormone and the precursor of the active metabolite 1,25-hydroxy vitamin D, otherwise known as calcitriol. Serum 25OHD concentration best reflects vitamin D nutritional status due to its long half-life and is a good predictor of bone quality in the elderly^26,27^. The causal relationship between 25OHD and musculoskeletal conditions remains controversial since previous epidemiological studies have shown conflicting results among individuals of different age stages. Some studies found a significant positive association between 25OHD and hip, femoral neck, or lumbar spine BMD in postmenopausal women^28–31^.Liu et al. showed a similar causal association in older adults^32^. Low serum 25OHD level was an independent risk factor for fragility fractures (e.g., hip fractures) in the elderly^33–35^. However, other studies did not reveal any associations between 25OHD and BMD or risk of fractures^36–40^.

Mendelian randomization analysis was applied to assess the association of lifelong circulating 25OHD concentration with BMD and found no causal relationship at heel, hip, femoral neck, or lumbar spine^22–24^. But it was necessary to conduct an up-to-date MR study to detect the causal link between 25OHD and BMD, because of (1) a large sample size of both 25OHD and BMD, (2) BMD summary data of cases in different age groups, (3) much more genetically associated SNPs, and (4) new MR analytical methods were available.

To conclude more robust, several analytical methods based on different assumptions of MR analysis were utilized with three groups of BMD summary GWAS data at different skeletal sites (FA-BMD, FN-BMD, and LS-BMD) and six groups of BMD summary GWAS data of different age stages (TB-BMD, TB-BMD (< 15), TB-BMD (15–30), TB-BMD (30–45), TB-BMD (45–60) and TB-BMD (> 60) ). The MR.RAPS method was carried out for its robustness to both systematic and idiosyncratic pleiotropy and weak instruments. The outlier variants identified by the MR-PRESSO outlier test were removed, and the global and distortion tests were also performed. The summary GWAS data we drew for 25OHD and BMD consisted uniquely (including 25OHD, FA-BMD, FN-BMF, LS-BMD, and TB-BMD) or mainly (TB-BMD in different age groups) of individuals of European descent and had been adjusted for many principal components, which would reduce potential bias. In sensitivity analysis, we observed some evidence of heterogeneity and directional pleiotropy in BMD subgroups, but the results from different methods were consistent. There was no potentially influential SNP driving the causal link under the “leave-one-out” analysis. To reduce the risk of pleiotropy, we repeated our analysis using SNPs in the four vitamin D genes (DHCR7, GC, CYP2R1, and CYP24A1) as an additional sensitivity analysis. In conclusion, no causal association was found between 25OHD and BMD either at different skeletal sites or across the lifespan, and the conclusion was reliable.

Reasons for this non-causal relationship might be interpreted as follows: Firstly, the effect of 25OHD on BMD might be determined by a combination of an increased plasma marker of bone resorption (C-telopeptide of type 1 collagen) and suppression of parathyroid hormone (PTH)–mediated bone turnover. High-dose vitamin D without extra calcium supplementation has been associated with an increase in calcitriol, which stimulates osteoclast genesis and differentiation^41–43^. Combined with a reduced PTH-mediated bone formation, it could even result in a dose-related decrease in volumetric BMD^44^. Secondly, the measurement of 25OHD in our GWAS did not show a sign of deficiency with a median, mean, and interquartile ranges of 47.9, 49.6, 33.5–63.2 nmol/L^25^. There was no evidence of associations between 25OHD and BMD without 25OHD deficiency. Thirdly, the measurement of BMD (g/cm^2^) of our included studies did not show a sign of osteoporosis, with participants from different age stages^45,46^. Associations between 25OHD and BMD might be weakened in the general population.

Our MR analysis showed that genetically lowered serum 25OHD concentration was not causally associated with BMD. Still, it would be reckless to deny the relationship between 25OHD and BMD and the role of vitamin D supplementation in the prevention and treatment of osteoporosis for targeted candidates. Clinical trials supported the skeletal benefits of vitamin D supplementation for persons with a serum 25OHD concentration < 30 nmol/L. At the same time, there was no benefit for untargeted community-dwelling older adults or healthy middle-aged men^47–49^. Hill et al. and Rebeca et al. suggested that consuming a vitamin D and calcium supplement improved 25OHD and BMD in sarcopenic older adults and postmenopausal women^50,51^. Besides, new parameters representing vitamin D sufficiency (e.g., bioavailable 25OHD) and bone quality (e.g., BMD measured by quantitative high-resolution computed tomography) may bring new light to the study of this relationship^52,53^.

Limitations still existed despite the MR design being less susceptible to bias from confounding factors and reverse causation than observational studies. First, potential pleiotropy could not be ruled out, but it was unlikely to change the conclusion in a clinically meaningful way. Second, population stratification might affect the association between variants and phenotypes, which was minimized by utilizing summary data from people uniquely or mainly of European ancestry in this study. However, care should be taken to extend our results beyond Europe. Third, the confidence intervals of the MR estimates were large, and the statistical powers were not satisfactory in age-stratified analysis for TB-BMD due to the small GWAS sample sizes for each of the age strata. These analyses were underpowered to exclude minor causal effects because the minimum detectable impact for 80% power was more than 0.05. Fourth, this study only tested the linear effect of vitamin D in the general population. Comprehensive studies such as one-sample MR analysis are needed to evaluate any nonlinear relationship between vitamin D and BMD in the epidemiologically high-risk population.

Our two-sample MR analysis did not support the causal effect of 25OHD on BMD at different skeletal sites across the lifespan. Updated randomized controlled trials are warranted to investigate the role of vitamin D supplementation in the prevention of osteoporosis in the high-risk populations.

## Methods

### 25OHD and BMD GWAS summary statistics

Details of the contributing GWAS consortiums are listed in Table 2. To obtain a more comprehensive and reliable conclusion of the causal link between 25OHD and BMD, we selected studies investigating traits related to 25OHD or BMD, having the largest sample sizes and consisting of the most similar populations while minimizing dataset overlap.

**Table 2:** Description of GWAS consortiums used for each phenotype.

Variable	First author (year)	Consortium	Sample size	Population	Adjustment
25OHD	Revez^25^	UKB	417,580	European	Sex, age, genotyping batch, assessment center, month of testing, supplement intake, and first 40 PCs
25OHD	Jiang^69^	SUNLIGHT	79,366	European	Sex, age, month of testing, BMI, and PCs
Forearm BMD	Zheng^45^ (2015)	GEFOS	10,805	European	Sex, age, and weight
Femoral neck BMD			49,988	European	
Lumbar spine BMD			44,731	European	
Total body BMD	Medina^46^	GEFOS	56,284	European	Age, weight, height, and genomic PCs
Total body BMD (0–15)			11,807	86%European	
Total body BMD (15–30)			4,180		
Total body BMD (30–45)			10,062		
Total body BMD (45–60)			18,805		
Total body BMD (> 60)			22,504		
Heel BMD	Kim^68^	UKB	394,929	European	Sex, age, and genotyping array

25OHD 25-hydroxyvitamin D, BMD bone mineral density, UKB UK Biobank, SUNLIGHT Study of Underlying Genetic Determinants of Vitamin D and Highly Related Traits, GEFOS Genetic Factors for Osteoporosis, PC principal components, BMI body mass index. There is no sample overlap between 25OHD (Revez) and Forearm/Femoral neck/Lumbar spine/Total body BMD. There is no sample overlap between 25OHD (Jiang) and Heel BMD.

We retrieved summary data for the association between SNPs and the serum 25OHD concentration from the UK Biobank (UKB) with phenotype, genotype, and clinical information on 417,580 individuals of European ancestry (age ranging from 40 to 69 years old)^25^. Serum 25OHD levels were measured quantitatively in blood samples collected from 2006 to 2013 by a chemiluminescent immunoassay (CLIA) in nmol/L. Participants with 25OHD concentrations below or above the validated range for the assay (10–375 nmol/L) were excluded. Individuals were identified by projecting the UKB sample to the first two principal components of the 1,000 Genome Project using Hap Map 3 SNPs with MAF > 0.01 in both data sets. Genotype data were quality-controlled and imputed to the Haplotype Reference Consortium and UK10K reference panels by the UKB group. Genetic variants with a minor allele count > 5 and imputation score > 0.3 for all individuals were extracted and converted genotype probabilities to hard-call genotypes using PLINK2. In total, 8,806,780 variants, including 260,713 SNPs on the X chromosome, were available for analysis. A linear mixed model GWAS was performed to identify the associations between genetic variants and the 25OHD concentration. Information regarding the quality control and statistical analyses has been reported previously^25^.

The femoral neck, lumbar spine, and forearm are the three common skeletal sites of postmenopausal women and men 50 years or older for measurement of BMD based on DXA. Total body BMD (TB-BMD) GWAS summary data is used to estimate the general effect of 25OHD on whole-body BMD. TB-BMD measurement is the most appropriate method for an unbiased assessment of BMD variation in the same skeletal site from childhood to old age^46^. GWAS summary statistics for BMD (unit, g/cm^2^) were downloaded from the GEFOS consortium (http://www.gefos.org/). We could also download GWAS summary statistics of BMD from the publicly available GWAS catalog website (https://www.ebi.ac.uk/gwas/downloads/summary-statistics) or the IEU GWAS database (https://gwas.mrcieu.ac.uk/datasets/). Three separate GWAS summary statistics of European participants’ femoral neck BMD (FN-BMD, n = 49,988), lumbar spine BMD (LS-BMD, n = 44,731), and forearm BMD (FA-BMD, n = 10,805) were downloaded from GEFOS; it was the largest GWAS on DXA-measured BMD to date^45^. A meta-analysis comprising 56,284 individuals of European ancestry was performed to investigate the genetic determinants of TB-BMD. The meta-analyzed effect size estimates were used in this study. Moreover, summary statistics for TB-BMD across the lifespan include 0–15 years (n = 11,807), 15–30 years (n = 4180), 30–45 years (n = 10,062), 45–60 years (18,805), and 60 or more years (n = 22,504), in which participants were mainly of the European ancestry (86%)^46^.

### Instrumental variables

From the GWAS summary data of 25OHD, we conducted a series of quality control steps to select eligible instrumental SNPs. Firstly, we extracted all SNPs that strongly and independently (R2 < 0.001) predicted exposure at genome-wide significance (*p* < 5E−08). Secondly, SNPs with a minor allele frequency (MAF) of < 0.01 were excluded to avoid the potential statistical bias from the original GWAS since they usually carry low confidence. Thirdly, extracting data for the above-selected SNPs from the outcome trait (BMD) GWAS summary. By default, if a particular requested SNP were not present in the outcome GWAS, then an SNP (proxy) that was in linkage disequilibrium (LD) (R2 > 0.8) from LDlink (https://ldlink.nci.nih.gov/) with the requested SNP (target) would be searched for instead. LD proxies were defined using 1000 genomes of European sample data. The effect of the proxy SNP on the outcome was returned, along with the proxy SNP, the effect allele of the proxy SNP, and the corresponding allele (in-phase) for the target SNP^54,55^. Fourthly, the effect of ambiguous SNPs with nonconcordant alleles (e.g., A/G vs. A/C) and palindromic SNPs with an ambiguous strand (i.e., A/T or G/C) was corrected, or the ambiguous and palindromic SNPs were directly excluded from the above-selected instrument SNPs in harmonizing process to ensure that the effect of an SNP on the exposure, and the effect of that same SNP on the outcome, corresponded to the same allele. These stringently selected SNPs were used as the instrumental variables for subsequent two-sample MR analysis.

According to the assumptions of MR analysis, the selected instrumental SNPs should be strongly associated with exposure. We calculated the F statistic to test whether there was a weak instrumental variable bias, namely, genetic variants selected as instrumental variables had a weak association with exposure. If the F statistic is much greater than 10 for the instrument-exposure association, the possibility of weak instrumental variable bias is small^56^.

### Mendelian randomization estimates

MR analysis uses genetic variants as instrumental variables to estimate the causative effect of exposure variables on an outcome. The study combined the summary statistics (β coefficients and standard errors) to estimate the causal associations between 25OHD and BMDs (including FN-BMD, LS-BMD, FA-BMD TB-BMD, and TB-BMD with different age groups) using different methods. Several robust methods have been proposed since it is unlikely that all genetic variants would be valid instrumental variables. The methods which included inverse variance weighted (IVW) with fixed effects (IVW-fixed) and random effects (IVW-random), MR-Egger regression, weighted median (WM), robust adjusted profile score (MR.RAPS) method, and MR-Pleiotropy RESidual Sum and Outlier (MR-PRESSO) method were based on different assumptions.

The IVW method uses a meta-analysis approach to combine Wald estimates for each SNP (i.e., the β coefficient of the SNP for BMD divides by the β coefficient of the SNP for 25OHD) to get the overall estimates of the effect of 25OHD on BMD^57^. If there is no violation of the IV2 assumption (no horizontal pleiotropy), or the horizontal pleiotropy is balanced, an unbiased causal estimate can be obtained by IVW linear regression^58,59^. Fixed and random effects IVW approaches are available. If significant heterogeneity (*P* < 0.05) is observed, a random-effect IVW model is applied.

If there is a particular direction of the horizontal pleiotropic effect, then constraining the slope to go through zero will introduce bias. Egger regression which allows the intercept to pass through a value other than zero, will relax the constraint. Based on the assumption of InSIDE, the MR-Egger regression performs a weighted linear regression of the outcome coefficients on the exposure coefficients^60^. Under the Instrument Strength Independent of Direct Effect (InSIDE) condition that instrument-exposure and pleiotropic effects are uncorrelated, it gives a valid test of the null causal hypothesis and a consistent causal effect estimate even when all the genetic variants are invalid IVs^61,62^.

The weighted median approach will provide an unbiased estimate of the causal effect in unbalanced horizontal pleiotropy even when up to 50% of SNPs are invalid IVs (e.g., due to pleiotropy)^63^.

MR-PRESSO is a method for detecting and correcting outliers in IVW linear regression. MR-PRESSO has three components, including (a) detection of horizontal pleiotropy (MR-PRESSO global test), (b) correction for horizontal pleiotropy via outlier removal (MR-PRESSO outlier test), and (c) testing of significant differences in the causal estimates before and after correction for outliers (MRPRESSO distortion test). The MR-PRESSO outlier test requires that at least 50% of the variants are valid instruments, have balanced pleiotropy, and rely on the InSIDE assumption^64^.

However, MR-Egger estimates may be inaccurate and strongly influenced by outlying genetic variants. The weighted median estimate, which does not require the InSIDE assumption, has been confirmed to have distinct superiorities over MR-Egger for its improved power of causal effect detection and lower type I error^63^. When the InSIDE assumption is valid, and the percentage of horizontal pleiotropic variants is small (≤ 10%), the causal estimate of the MR-PRESSO outlier adjustment is less biased and has better precision (smaller standard deviation) than MR-Egger. However, when the percentage of horizontal pleiotropic variants is high (≥ 50%), the opposite is found^64^. The weighted median has less bias and less precision in the causal estimate than the MR-PRESSO outlier test, mainly when the percentage of horizontal pleiotropic variants is < 50%^64^.

Since we might include weak instrumental variables in the analyses, we developed a recently proposed method called robust adjusted profile score (MR.RAPS) to make our results more reliable^65^. This method is robust to systematic and idiosyncratic pleiotropy and can give a robust inference for MR analysis with many weak instruments. It can correct for pleiotropy using robust adjusted profile scores and is recommended to routinely use the RAPS estimator in practice, especially if the exposure and the outcome are both complex traits.

Analyses were performed using R version 4.1.2 (R Foundation for Statistical Computing, Vienna, Austria) using the TwoSampleMR R package of MR-Base (https://github.com/MRCIEU/TwoSampleMR)^58^. The results reported the effect size as the effect of a one-standard-deviation (1-SD) change in rank-based inverse normal transformed (RINT) 25OHD level. Results were considered insignificant if *P* values ≥ 0.05 for all MR methods. If the estimates of different methods were inconclusive, the link between exposure and outcome phenotype with an adjusted *P* value < 0.05/5 = 0.01 (Bonferroni correction for multiple testing) was considered significant.

### Sensitivity analyses

We conducted the IVW and MR-Egger^66^ methods to detect heterogeneity. The Cochran’s Q statistic quantified heterogeneity, and a *P* value of < 0.05 would be regarded as significant heterogeneity. To identify potentially influential SNPs, we performed a “leave-one-out” sensitivity analysis. We excluded one SNP at a time and performed an IVW-random method on the remaining SNPs to identify the potential influence of outlying variants on the estimates.

The intercept of the MR-Egger regression line was used to quantify the amount of horizontal pleiotropy present in the data averaged across the genetic instruments^61,62^. Under the InSIDE assumption, the MR-Egger intercept test identifies directional horizontal pleiotropy if the intercept from the MR-Egger analysis is not equal to zero^62^.

We further applied the MR-PRESSO global test^64^ to reduce heterogeneity in estimating the causal effect of removing SNPs that were horizontal pleiotropic outliers. We conducted this analysis using the MR-PRESSO R package (https://github.com/rondo lab/MR-PRESSO). The number of distributions was set to 1000, and the threshold was set to 0.05.

When selecting SNPs from a very large GWAS, it can be challenging to determine whether an SNP satisfies the second and third MR assumptions. To reduce the risk of pleiotropy, we repeated our analyses using SNPs in or near genes (DHCR7, GC, CYP2R1, and CYP24A1) encoding enzymes and carrier proteins involved in vitamin D synthesis or metabolism as instrumental variables.

### Multivariable MR analysis

One advantage of multivariable MR (MVMR) analysis compared to univariable MR is that SNPs that are thought to affect multiple exposures potentially, or where it is not clear exactly which exposure they affect, can be included when estimating the effects of the exposures on the outcome. This makes MVMR particularly useful when the exposures are closely related, or one (or more) is thought to be a potential pleiotropic pathway from the SNPs to the outcome. Meanwhile, MVMR does not introduce collider bias into the results. Because of a solid phenotypic association between vitamin D and BMI, we included BMI in an MVMR analysis to explore the direct effect of vitamin D on BMD after adjusting for BMI. Summary-level data for BMI was drawn from a meta-analysis comprising 322,154 individuals of European ancestry from the Genetic Investigation of Anthropometric Traits (GIANT) consortium^67^, without sample overlap with our 25OHD GWAS.

### Replication analysis

To increase the power of analysis in our study, the GWAS summary statistics for ultrasound-measured heel BMD were also drawn from the UKB, including 394,929 European participants^68^. While performing a two-sample MR analysis where both effects for exposure and outcome come from the same population might induce weak instrument bias or bias due to winners’ curse, we extracted effects of the above-selected SNPs from the SUNLIGHT consortium GWAS including 79,366 European participants and studied the association^69^.

### Power assessment

We estimated the power of our study according to a method suggested by Brion et al.^70^. The equations use an approximate linear model, which requires the proportion of phenotypic variation explained by IV SNPs, the effect size of the exposure to the outcome at the epidemiological level, sample size, and the standard deviation (SD) of the exposure and outcome.

### Procedures of MR analysis

Our study first performed MR analysis with all the above-selected SNPs as IVs. If the MR-PRESSO analysis detected a significant horizontal pleiotropy, we removed the outlier variants (with a *P* value less than the threshold in the MR-PRESSO outlier test) and performed MR analysis again. After the MR-PRESSO outlier removal step, if the heterogeneity was still significant, we would perform MR analysis under the condition of removing all the SNPs. The *P* value was less than 1 in the MR-PRESSO outlier test. At last, if potentially influential SNPs were identified in the “leave-one-out” sensitivity analysis, we should conclude with caution.

### Ethics

Our analysis used published studies or publicly available GWAS summary data. No original data was collected for this manuscript, and thus, no ethical committee approval was required. Each study included was approved by their institutional ethics review committees, and written informed consent was provided by all participants or by their parents in the case of children.

## Supplementary Information

Supplementary Information 1.

Supplementary Information 2.

## Data Availability

The datasets supporting the conclusions of this article are publicly available. The other data generated or analyzed during this study are available in this published article and its Supplementary file.

## Abbreviations

BMD: Bone mineral density
BMI: Body mass index
CI: Confidence interval
DXA: Dual-energy X-ray absorptiometry
FA: Forearm
FN: Femoral neck
GEFOS: GEnetic Factors for OSteoporosis Consortium
GWAS: Genome-wide association study
InSIDE: Instrument Strength Independent of Direct Effect
ID: Identification
IEU: Integrative Epidemiology Unit
IV: Instrument variable
IVW: Inverse variance weighted
LD: Linkage disequilibrium
LS: Lumbar spine
MAF: Minor allele frequency
MR: Mendelian randomization
MR-PRESSO: MR-Pleiotropy RESidual Sum and Outlier
MR.RAPS: MR Robust Adjusted Profile Score
MVMR: Multivariable Mendelian randomization
RCT: Randomized controlled trial
SD: Standard deviation
SE: Standard error
SNP: Single-nucleotide polymorphism
UKB: UK biobank
TB: Total body
